# The associations of obesity phenotypes with the risk of hypertension and its transitions among middle-aged and older Chinese adults

**DOI:** 10.4178/epih.e2023043

**Published:** 2023-04-10

**Authors:** Ziyue Sheng, Shang Lou, Jin Cao, Weidi Sun, Yaojia Shen, Yunhan Xu, Ziyang Ren, Wen Liu, Qian Yi, Peige Song

**Affiliations:** 1 School of Public Health, Women’s Hospital, Zhejiang University School of Medicine, Hangzhou, China; 2 School of Public Health, Department of Maternal and Child Health, Health Science Centre, Peking University, Beijing, China

**Keywords:** Obesity, Central obesity, Hypertension, Sex characteristics, Asian

## Abstract

**OBJECTIVES:**

This study aimed to investigate the associations of obesity phenotypes with hypertension stages, phenotypes, and transitions among middle-aged and older Chinese.

**METHODS:**

Using the 2011-2015 waves of the China Health and Retirement Longitudinal Study, we conducted a cross-sectional analysis included 9,015 subjects and a longitudinal analysis included 4,961 subjects, with 4,872 having full data on the hypertension stage and 4,784 having full data on the hypertension phenotype. Based on body mass index and waist circumstance, subjects were categorized into 4 mutually exclusive obesity phenotypes: normal weight with no central obesity (NWNCO), abnormal weight with no central obesity (AWNCO), normal weight with central obesity (NWCO), and abnormal weight with central obesity (AWCO). Hypertension stages were classified into normotension, pre-hypertension, stage 1 hypertension, and stage 2 hypertension. Hypertension phenotypes were categorized as normotension, pre-hypertension, isolated systolic hypertension (ISH), isolated diastolic hypertension (IDH), and systolic-diastolic hypertension (SDH). The association between obesity phenotypes and hypertension was estimated by logistic regression. A comparison between different sexes was conducted by testing the interaction effect of sex.

**RESULTS:**

NWCO was associated with normal→stage 2 (odds ratio [OR], 1.95; 95% confidence interval [CI], 1.11 to 3.42), maintained stage 1 (OR, 1.62; 95% CI, 1.14 to 2.29), and normal→ISH (OR, 1.39; 95% CI, 1.05 to 1.85). AWCO was associated with normal→stage 1 (OR, 1.75; 95% CI, 1.40 to 2.19), maintained stage 1 (OR, 2.77; 95% CI, 2.06 to 3.72), maintained stage 2 (OR, 2.80; 95% CI, 1.50 to 5.25), normal→ISH (OR, 1.56; 95% CI, 1.20 to 2.02), and normal→SDH (OR, 2.54; 95% CI, 1.72 to 3.75). An interaction effect of sex existed in the association between obesity phenotypes and hypertension stages.

**CONCLUSIONS:**

This study highlights the importance of various obesity phenotypes and sex differences in hypertension progression. Tailored interventions for different obesity phenotypes may be warranted in hypertension management, taking into account sex-specific differences to improve outcomes.

## INTRODUCTION

Hypertension is a major public health problem that poses a considerable burden on economies and society. The global prevalence of hypertension has increased substantially over time, with an estimated 22.0% of the world’s population affected by hypertension in 2014 and increased to 32% in 2019 [1,2]. In China, the prevalence of hypertension reached 27.9% in 2015 [3], and further increased to 31.4% in 2022 [1]. Notably, hypertension has been shown to increase the risk of death from chronic kidney disease, stroke, coronary heart disease, and pulmonary heart disease [4,5]. Therefore, it is crucial to identify and mitigate potential risk factors early on, promoting the prevention of the onset and exacerbation of hypertension through effective interventions.

Obesity has emerged as the leading risk factor for hypertension, with body mass index (BMI) being the primary measure of obesity used in most studies and public health interventions [3,6-9]. However, waist circumference (WC) is a more reliable measure than BMI for capturing visceral fat [9]. Combining BMI and WC enables a more nuanced classification of obesity phenotypes, facilitating the exploration of the independent and combined effects of general and central obesity. For example, the normal weight central obesity phenotype (NWCO, with normal BMI and an excessive WC) highlights the independent effect of central fat distribution. On the other hand, the abnormal weight and central obesity phenotype (AWCO, abnormal BMI and excessive WC) captures the combined effects of abnormal weight and central obesity.

The classification of hypertension typically relies on systolic blood pressure (SBP) and diastolic blood pressure (DBP). Based on pathophysiology, hypertension can be classified into four distinct phenotypes [3]: normotension, isolated systolic hypertension (ISH), isolated diastolic hypertension (IDH), and systolic-diastolic hypertension (SDH), each representing a unique pathophysiological profile. For example, patients with ISH tend to have severer arterial stiffness, while those with IDH are predisposed to higher peripheral vascular resistance [10]. Given the diverse phenotypes of both obesity and hypertension, the relationship between these two health conditions should be explored through a comprehensive analysis of the phenotypes of both obesity and hypertension.

Existing studies have yielded incongruent results concerning the association between obesity and hypertension. Some studies have demonstrated that a higher level of visceral fat is associated with an increased risk of metabolic disorders, leading to heightened blood pressure [11,12]. Nevertheless, a cohort study suggested that people with general obesity had a higher risk of developing hypertension than those with NWCO [13]. Hypertension severity is typically divided into four levels (normal, and stage 1, 2, 3 hypertension), reflecting a spectrum of blood pressure levels from low to high [3]. However, few studies have elucidated the effect of distinct obesity phenotypes on hypertension status and phenotypes, as well as their respective contribution to hypertension progression. A more comprehensive understanding of how obesity phenotypes affect hypertension stages and phenotypes is necessary to inform early-stage intervention strategies for hypertension management.

To address these gaps in literature, this study aimed: (1) to investigate the association of obesity phenotypes with different stages and phenotypes of hypertension in a cross-sectional design; and (2) to explore the associations of different obesity phenotypes with the transitions of hypertension stages and phenotypes in a longitudinal design.

## MATERIALS AND METHODS

### Study population

All participants included in this study were from the China Health and Retirement Longitudinal Study (CHARLS), a large-scale interdisciplinary survey project conducted by the Peking University National School of Development. The CHARLS adopted a stratified four-stage sampling method, covering 150 districts and counties in 28 provinces across China [14-16]. Selected participants were all aged 45 years or above. The nationwide baseline survey was launched in 2011 to collect information on socioeconomic and health-related conditions through a face-to-face computer-assisted personal interview. Respondents were followed up biennially thereafter.

**Figure 1.**
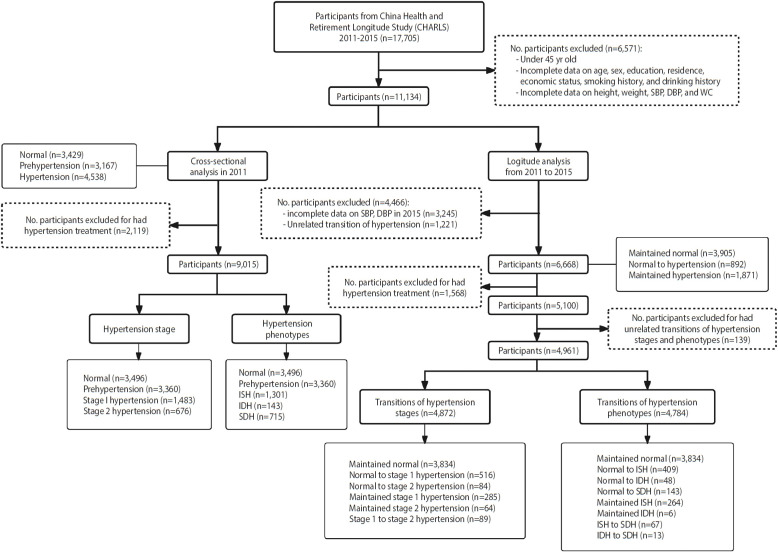
Flowchart of the study. SBP, systolic blood pressure; DBP, diastolic blood pressure; WC, waist circumstance; ISH, isolated systolic hypertension; IDH, isolated diastolic hypertension; SDH, systolic diastolic hypertension.

In this study, we used data from the CHARLS 2011-2015 [15]. Our cross-sectional analysis used data from 2011 baseline, while the longitudinal analysis used data from 2011 and 2015; The sensitivity analysis used participants’ data from 2013 and 2015 (Figure 1).

In the cross-sectional analysis to explore the association of obesity phenotypes with hypertension stages and phenotypes, we excluded individuals on antihypertensive medication (n= 2,119) or with incomplete data on sex, education, economic status, smoking history, SBP, DBP, height, weight, and WC (n= 6,571) from CHARLS 2011. Consequently, 9,015 participants were remained for analysis.

In the longitudinal analysis examining the associations between obesity phenotypes and transitions of hypertension stages and phenotypes, the follow-up time was standardized to four years. Participants with incomplete data on hypertension transitions, or those who exhibited reverse transitions in hypertension stages and phenotypes (i.e., from hypertension to normal or transition from a more severe to a milder status) were excluded. This led to the exclusion of 228 participants for hypertension stage analysis and 316 for hypertension phenotype analysis. Consequently, 4,872 participants were retained in the analysis of transitions between hypertension stages, and 4,784 in the analysis of transitions between hypertension phenotypes.

### Assessment of hypertension and transitions between hypertension stages and phenotypes

Participants’ blood pressure (BP) was measured on the left arm, with the procedure repeated for three times at a 45-second interval [16]. The average value of the three measurements was calculated and rounded to the nearest 1 mmHg. Hypertension stages were defined based on SBP and DBP as follows: normal (SBP<140 mmHg and DBP< 90 mmHg), prehypertension (SBP 120-139 mmHg or DBP 80-89 mmHg), stage 1 hypertension (SBP 140-159 mmHg or DBP 90-99 mmHg), stage 2 hypertension (SBP 160-179 mmHg or DBP 100-109 mmHg), and stage 3 hypertension (SBP≥180 mmHg or DBP≥ 110 mmHg). Due to the limited sample size, stage 3 hypertension was combined with stage 2 hypertension in this study. Hypertension phenotypes were divided into 4 categories: normal (SBP< 140 mmHg and DBP< 90 mmHg), ISH (SBP≥ 140 mmHg and DBP<90 mmHg), IDH (SBP<140 mmHg and DBP≥90 mmHg), and SDH (SBP≥ 140 mmHg and DBP≥ 90 mmHg).

Hypertension transition was assessed by comparing participants’ hypertension status at baseline in 2011 and at the endpoint in 2015. Hypertension stage transitions were categorized as follows: maintained normal, normal to stage 1 hypertension, normal to stage 2 hypertension, maintained stage 1 hypertension, maintained stage 2 hypertension, and stage 1 to stage 2 hypertension. Hypertension phenotype transitions were classified as: maintained normal, normal to ISH, normal to IDH, normal to SDH, ISH to SDH, and IDH to SDH.

### Assessment of anthropometry indices and definition of obesity phenotypes

Participants’ height and weight were measured to the nearest 0.1 m and 0.1 kg respectively. Height was measured with participants wearing slippers and standing upright on the height meter pedal with their backs against the vertical column. The feet were evenly weighted. The heels were close together and close to the vertical board. Body weight was measured after participants removed shoes, heavy coats, and any heavy objects from their pockets [16]. BMI was calculated as weight (kg) divided by height squared (m^2^) [7,17]. According to the 2018 Chinese guidelines for hypertension management, participants were divided into four stages of obesity [18]: underweight (BMI< 18.5 kg/m^2^), normal (18.5 kg/m^2^ ≤ BMI< 24.0 kg/m^2^), overweight (24.0 kg/m^2^ ≤ BMI < 28.0 kg/m^2^), and obesity (BMI≥ 28.0 kg/m^2^). Overweight and obesity were considered as abnormal weight. Underweight and normal weight were combined into the normal group in this study because this study primarily focused on the effect of excess body weight on hypertension. WC was measured at the level of the navel when participants held their breath at the end of exhalation [16]. Males with a WC≥ 90 cm and females with a WC≥ 85 cm were classified as having excessive WC, indicating central obesity [18]. Participants were divided into four groups according to both BMI and WC: normal weight with no central obesity (NWNCO), abnormal weight with no central obesity (AWNCO), NWCO, and AWCO.

### Covariates

Relevant covariates, including age, sex, residence, educational level, economic status, smoking history, and drinking history, were collected through self-reported questionnaires at baseline. Educational level was divided into four levels: never received a formal education, primary school, middle school, and high school and above. Economic status was assessed by the natural logarithm of per capita expenditures [19], which included the costs of communication, utilities, fuels, a housekeeper, local transportation, household items, and entertainment. Subsequently, participants were divided into low, middle, and high economic status by tertiles of the natural logarithm of per capita expenditures. Residence was defined by the location of participants and was divided into urban and rural.

### Statistical analysis

In the descriptive analysis, continuous variables with a normal distribution were presented using mean and standard deviation (SD), while those exhibiting a skewed distribution were reported using medians with interquartile ranges (IQR). Categorical variables were summarized using numbers with percentages (%). Inter-group comparisons were conducted using analysis of variance for continuous variables with normal distribution and the rank sum test for those with skewed distributions. For categorical variables, the chi-square test was conducted.

General logistic regression models were performed to examine the association between obesity phenotypes and hypertension stages and phenotypes in the cross-sectional analysis, as well as the association of obesity phenotypes with transitions of hypertension stages and phenotypes in the longitudinal analysis. Associations were reported as odds ratios (ORs) with 95% confidence intervals (CIs). To assess the interaction effect between sex and obesity phenotype, an interaction item for obesity phenotype and sex was added in the logistic regression models. A significant association between the interaction item and hypertension-related outcomes suggested the existence of an interaction effect, and a subsequent sex-stratified analysis of the associations was conducted.

To estimate the independent effect of elevated BMI, an additional logistic regression model was performed to calculate the OR (95% CI) for one-unit increment in BMI, with further stratification by central obesity (defined by WC). Similarly, to estimate the independent effect of excessive WC, the OR (95% CI) of one-unit increment in WC was calculated and stratified by general obesity defined by BMI. The corresponding results can be found in Supplementary Materials 1 and 2.

All models were adjusted for age, sex, residence, educational level, economic status, smoking history, and drinking history. In the sensitivity analysis, participants with complete data in both 2013 and 2015 were included, using the same analysis approach in the longitudinal analysis. Analyses were conducted using SAS version 9.4 (SAS Institute Inc., Cary, NC, USA). The tests were 2-sided, with a significance level of p-value< 0.05.

### Ethics statement

The CHARLS was approved by the Peking University Biomedical Ethics Committee (IRB approval No. IRB00001052-11015 for the main household survey and IRB00001052-11014 for biomarker collection). All respondents had signed informed consent forms.

## RESULTS

### Characteristics of participants in the cross-sectional study (2011)

Among all participants, 49.4% were male and 50.6% were female, and the mean age was 58.46± 9.33 years. The mean WC was 84.12± 9.53 cm, and the mean BMI was 23.00± 3.37 kg/m^2^. The numbers of participants categorized as NWNCO, AWNCO, NWCO, and AWCO were 4,029, 208, 1,793, and 2,985, respectively. The mean SBP was 127.21± 19.77 mmHg, and the mean DBP was 74.57± 11.53 mmHg (Table 1).

### Associations of obesity phenotypes with hypertension stages and phenotypes with sex disparities in 2011

Using NWNCO as the reference group, AWNCO (OR, 1.69; 95% CI, 1.06 to 2.69), NWCO (OR, 1.61; 95% CI, 1.35 to 1.92), and AWCO (OR, 3.06; 95% CI, 2.62 to 3.57) were all associated with stage 1 hypertension. Both NWCO (OR, 2.01; 95% CI, 1.58 to 2.55) and AWCO (OR, 3.53; 95% CI, 2.84 to 4.39) were associated with stage 2 hypertension. As for hypertension phenotypes, AWNCO was associated with SDH (OR, 2.10; 95% CI, 1.17 to 3.78), NWCO was associated with SDH (OR, 1.87; 95% CI, 1.47 to 2.39) and ISH (OR, 1.63; 95% CI, 1.35 to 1.97), and AWCO was associated with all 3 hypertension phenotypes (OR for ISH, 2.58; 95% CI, 2.17 to 3.07; OR for IDH, 4.08; 95% CI, 2.72 to 6.12; OR for SDH, 4.19, 95% CI, 3.42 to 5.13).

**Figure 2.**
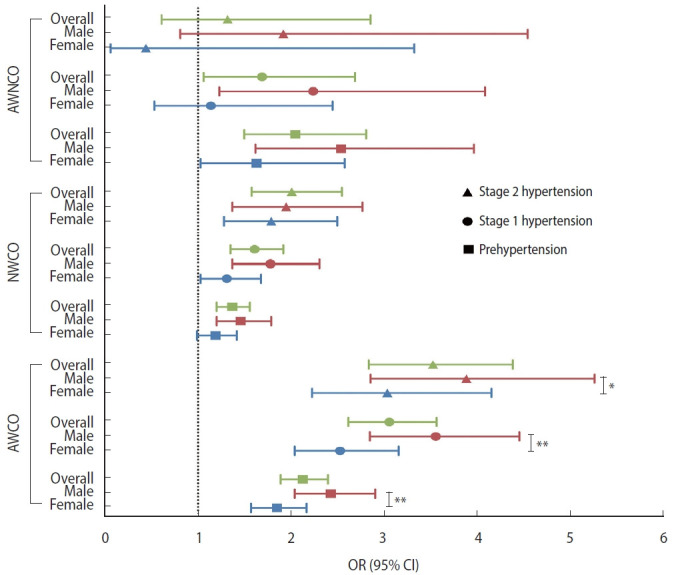
Forest plot of the associations between obesity phenotypes and hypertension stages in cross-sectional analysis. AWNCO, abnormal weight non-central obesity; NWCO, normal weight central obesity; AWCO, abnormal weight central obesity; OR, odds ratio; CI, confidence interval. *p<0.05, **p<0.01.

**Figure 3.**
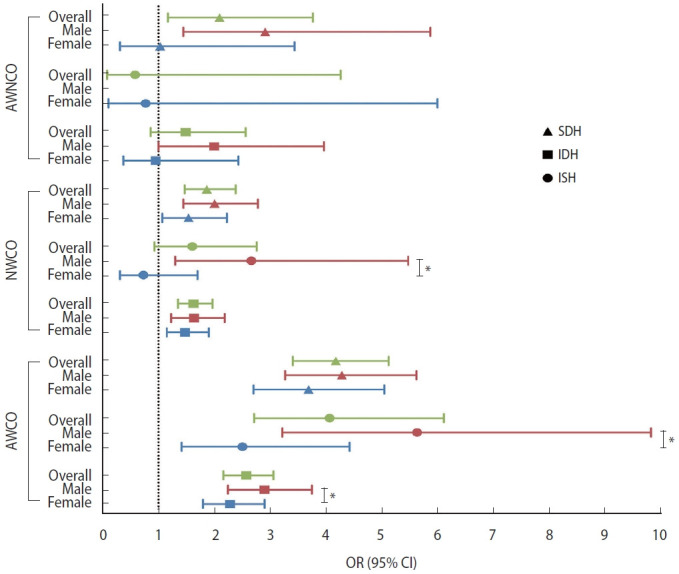
Forest plot of the associations between obesity phenotypes and hypertension phenotypes in cross-sectional analysis. AWNCO, abnormal weight non-central obesity; NWCO, normal weight central obesity; AWCO, abnormal weight central obesity; SDH, systolic diastolic hypertension; IDH, isolated diastolic hypertension; ISH, isolated systolic hypertension; OR, odds ratio; CI, confidence interval. *p<0.05.

Sex had an interaction effect with AWCO on the associations with prehypertension, stage 1 hypertension, and stage 2 hypertension (Figure 2), as well as with NWCO on the association with IDH, and with AWCO on the associations with IDH and ISH (Figure 3). Upon stratification by sex, the association of AWCO with stage 1 hypertension (p-interaction< 0.01) and stage 2 hypertension (p-interaction< 0.05) was stronger in males than in females (OR in males, 2.24; 95% CI, 1.23 to 4.09; OR in females, 1.14; 95% CI, 0.53 to 2.45). The association of NWCO with IDH was significant in males but not in females (OR in males, 2.67; 95% CI, 1.30 to 5.48; OR in females, 0.73; 95% CI, 0.31 to 1.70), and the associations of AWCO with ISH and IDH were stronger in males (OR for ISH, 2.91; 95% CI, 2.25 to 3.76; OR for IDH, 5.64; 95% CI, 3.23 to 9.85) than in females (OR for ISH, 2.29; 95% CI, 1.80 to 2.91; OR for IDH, 2.51; 95% CI, 1.42 to 4.44) (p-interaction< 0.05) (Table 2).

### Characteristics of participants in the longitudinal study (2011 to 2015)

Among the 4,961 included participants, 2,378, 122, 968, and 1,493 were categorized as NWNCO, AWNCO, NWCO, and AWCO, respectively. Participants’ mean age was 61.57± 8.65 years. The mean WC was 83.32± 9.20 cm and the mean BMI was 22.81±3.25 kg/m^2^. The mean SBP and DBP were 124.68± 18.70 mmHg and 73.38± 10.85 mmHg, respectively (Table 3).

### Associations of obesity phenotypes with the transitions of hypertension stages and phenotypes from 2011 to 2015

Compared with participants with NWNCO, those with NWCO had a higher risk of a transition from normal to stage 2 hypertension (OR, 1.95; 95% CI, 1.11 to 3.42) and maintained stage 1 hypertension (OR, 1.62; 95% CI, 1.14 to 2.29). AWCO was significantly associated with the transitions from normal to stage 1 hypertension (OR, 1.75; 95% CI, 1.40 to 2.19), maintained stage 1 hypertension (OR, 2.77; 95% CI, 2.06 to 3.72) and maintained stage 2 hypertension (OR, 2.80; 95% CI, 1.50 to 5.25). However, AWNCO was not significantly associated with any type of hypertension stage transition (Supplementary Material 3). As for hypertension phenotype transitions, compared with NWNCO, AWNCO (OR, 1.99; 95% CI, 1.09 to 3.62) and NWCO (OR, 1.39; 95% CI, 1.05 to 1.85) were associated with the transition from normal to ISH, and AWCO was associated with the transitions from normal to ISH (OR, 1.56; 95% CI, 1.20 to 2.02) and from normal to SDH (OR, 2.54; 95% CI, 1.72 to 3.75) (Supplementary Material 4). The results are demonstrated in Table 4.

The interaction effect of sex and obesity phenotypes was not significant in all hypertension stage transitions and phenotype transitions (p-interaction> 0.05).

## DISCUSSION

This study explored the associations of distinct obesity phenotypes with hypertension stages and phenotypes, as well as transitions of hypertension stages and phenotypes. The study found that both general obesity and central obesity were associated with hypertension. Notably, when focusing on the transitions of hypertension stages, general obesity was not associated with any hypertension stage transitions, whereas central obesity and a combination of general obesity and central obesity were associated with the progression or persistence of hypertension. For hypertension phenotypes, both central obesity and general obesity, as well as a co-existence of these two statuses, were associated with SDH. A higher risk of ISH was found in participants with central obesity, while a higher risk of IDH was only significant in participants with both abnormal weight and WC. The risk from obesity phenotypes displayed a sex disparity as the association between central obesity and hypertension phenotypes was stronger in males than in females.

### Associations of both general obesity and central obesity with hypertension

Our findings are consistent with previous studies that demonstrated a significant association between general obesity and hypertension [20,21], as well as similar associations for central obesity [11,12,22,23]. For example, several studies conducted in China found that NWCO was associated with hypertension [12,22]. Furthermore, some studies conducted in Western populations also demonstrated the association between a greater WC or visceral fat and hypertension [23,24]. Importantly, different hypertension phenotypes have distinct pathophysiology. Specifically, ISH indicates arterial stiffness, and the relationship between obesity and ISH is usually attributed to the sympathetic nervous system, which can cause norepinephrine spillover and oxidative stress that can damage vascular elasticity and ultimately lead to an elevation of SBP [18,25]. Meanwhile, IDH indicates higher peripheral vascular resistance [10]. However, due to the small proportion of IDH patients among all hypertension patients and the fact that many previous studies did not analyze IDH as a risk factor for cardiovascular disease (CVD) [26,27], research focused on the mechanisms of obesity on IDH incidence is limited. Although recent studies have reported an association between IDH and CVD [28], more research is needed to explore the underlying mechanisms of IDH occurrence, as well as the role that obesity in it.

### The associations of different obesity phenotypes with transitions between hypertension stages and phenotypes

The association between obesity and hypertension has been validated in numerous studies, but little is known about the effect of obesity phenotypes on the transitions of hypertension stages and phenotypes. In our study, central obesity, but not general obesity, was associated with hypertension stage transitions. We also found that participants with central obesity were more prone to developing ISH than IDH. This finding aligns with a study conducted in Algeria, which reported that only DBP was negatively associated with the waist-to-hip ratio (WHR) [21], while SBP was not associated with any traditional anthropometric indicators (BMI, WC, and WHR). Research exploring underlying mechanisms indicates that people with central obesity are prone to have ISH rather than IDH or SDH. The essential mechanism of the maintenance and amplification of obesity-related chronic inflammation may be the local cytokine crosstalk between adipocytes and immune cells [29]. The accumulation of fat in the abdominal area and visceral stores promotes the inflammation of local adipose tissue, resulting in the release of immune-related adipokines, which are related to endogenous activation and atherosclerosis, and at the same time can lead to increased blood pressure through non-proinflammatory pathways [29]. In obesity, upregulated resistin in adipose tissue inhibits endothelial nitric oxide synthase (eNOS) activity by increasing the expression of phosphatase and tensin homolog deleted on chromosome 10 (PTEN), but some protein hormones, such as adiponectin, are downregulated [30]. Adiponectin can activate adenosine monophosphate-activated protein kinase and eNOS in endothelial cells, whereas free fatty acids produced by adipose tissue can inhibit the activity of eNOS, block the insulin-mediated nitric oxide synthesis pathway, and thus weaken its vasodilation effect [29].

Furthermore, our results showed that general obesity contributed to the transition from normal to ISH. This finding is incongruent with the study conducted in Algeria [21]. However, the incongruence may be due to differences in study design. For instance, the study in Algeria was cross-sectional, included a smaller sample size (n= 785), and used its own anthropometric cut-offs rather than following the World Health Organization’s criteria or national criteria. Furthermore, genetic factors and the early-life environment may have also contributed to disparity in results of those two studies [31].

We also found that, compared to NWNCO, participants with central obesity or both central obesity and general obesity had higher risks of maintained hypertension or hypertension progression, whereas that association was not found in participants with general obesity. This finding aligns with results from several cohort studies [32], highlighting the importance of integrating these two independent indicators in early-stage screening and prevention of hypertension. Current guidelines on hypertension prevention primarily focus on BMI or WC alone, but the negative impact of the simultaneous presence of general obesity and central obesity should not be overlooked, as indicated by the results of this study.

### Sex disparities in the effect of obesity phenotypes

Sex was found to have an interaction effect with obesity phenotypes in the associations with hypertension stages and phenotypes, but not in the associations with transitions between stages and phenotypes. To be specific, the associations of NWCO and AWCO with hypertension were stronger in males stronger than in females.

The sex disparity could be attributed to both physiological and lifestyle factors, as females are less likely to engage in lifestyles that pose risks to health, such as smoking and drinking [12]. However, many other studies found contrasting results compared with the current study [21,22,33]. Nevertheless, most of those studies were cross-sectional, and this incongruency may derive from hypertension duration, genetic factors, or the early-life environment, as mentioned above [31]. To clarify the mechanisms underlying the sex difference, more studies, especially cohort studies, are warranted.

Moreover, due to the absence of sex disparities in the association of AWNCO with hypertension stages and phenotypes, it is reasonable to conjecture that some sex-related physiological properties are involved in the association of central obesity with hypertension, but not with the association of general obesity. Physiological and psychological factors that differ between the sexes deserve more attention in future research on the mechanisms of how central obesity or general obesity influences blood pressure.

To the best of our knowledge, this study is the first to explore the association between obesity phenotypes and the development of hypertension stages and phenotypes, and to conduct analyses from both cross-sectional and longitudinal perspectives, thereby providing deeper insights into the associations of obesity phenotypes with hypertension. By comparing different obesity phenotypes (NWNCO, AWNCO, NWCO, and AWCO), our study illuminated the singular and combined effects of general obesity and central obesity. This approach underscores the necessity of integrating both BMI and WC in early-stage screening and prevention of hypertension, since our study showed that people with normal weight but with excessive WC (NWCO) are predisposed to developing hypertension, and people possessing both abnormal weight and excessive WC are at a higher risk. In addition, the sex-stratified results indicated potential mechanistic discrepancies between the sexes and also highlighted the importance of specific prevention and treatment strategies for targeted high-risk populations.

This work also has several limitations. First, participants who had used antihypertension drugs were excluded. The excluded population had a larger proportion of central obesity and more severe levels of hypertension than the retained population, as well as a significant difference in phenotypes, all of which may have resulted in selection bias. This also rendered it more difficult for the analysis to detect possible associations. Second, the IDH group had a small sample size; therefore, the estimation for the association of obesity phenotypes with developing IDH may have been somewhat inaccurate. Third, we used obesity data in 2011 to determine exposure, and used hypertension data in 2011 and 2015 to determine outcomes, meaning that subjects’ exposure and outcome may have overlapped. Thus, we conducted a sensitivity analysis using hypertension data from 2013 and 2015. The results indicated that our analysis was robust (Supplementary Material 5).

This study validated the association between obesity phenotypes and hypertension development and its transition. The effects of different obesity phenotypes were explored, and the study found that the combination of abnormal weight and central obesity was significantly associated with hypertension stages, phenotypes, and transitions, and therefore deserves more attention in early-stage screening and intervention. This study also discovered sex disparities in the association of obesity phenotypes with hypertension. Overall, more longitudinal studies are needed to explore the mechanism underlying different obesity phenotypes in transitions between hypertension stages and phenotypes, as well as sex differences.

## Figures and Tables

**Table 1.** Characteristics of participants in 2011 according to obesity phenotype

Characteristics		Overall (n=9,015)	NWNCO (n=4,029)	AWNCO (n=208)	NWCO (n=1,793)	AWCO (n=2,985)	p-value
Age (yr)		58.46±9.33	59.27±9.45	56.00±8.51	60.24±9.84	56.47±8.48	<0.001
Sex							<0.001
	Male	4,457 (49.4)	2,514 (62.4)	118 (56.7)	668 (37.3)	1,157 (38.8)	
	Female	4,558 (50.6)	1,515 (37.6)	90 (43.3)	1,125 (62.7)	1,828 (61.2)	
Educational level							<0.001
	No formal education	4,143 (46.0)	1,915 (47.5)	80 (38.5)	911 (50.8)	1,237 (41.4)	
	Primary school	2,011 (22.3)	941 (23.4)	49 (23.6)	369 (20.6)	652 (21.8)	
	Middle school	1,865 (20.7)	789 (19.6)	48 (23.1)	327 (18.2)	701 (23.5)	
	High school and above	996 (11.1)	384 (9.5)	31 (14.9)	186 (10.4)	395 (13.2)	
Economic status^1^							<0.001
	Low	2,973 (33.0)	1,462 (36.3)	67 (32.2)	604 (33.7)	840 (28.1)	
	Middle	3,013 (33.4)	1,388 (34.5)	64 (30.8)	595 (33.2)	966 (32.4)	
	High	3,029 (33.6)	1,179 (29.3)	77 (37.0)	594 (33.1)	1,179 (39.5)	
Residence							<0.001
	Rural	5,949 (66.0)	2,949 (73.2)	132 (63.5)	1,136 (63.4)	1,732 (58.0)	
	Urban	3,066 (34.0)	1,080 (26.8)	76 (36.5)	657 (36.6)	1,253 (42.0)	
Smoking history		3,724 (41.3)	2,100 (52.1)	84 (40.4)	580 (32.4)	960 (32.2)	<0.001
Drinking history		2,388 (26.5)	1,280 (31.8)	52 (25.0)	394 (22.0)	662 (22.2)	<0.001
SBP (mmHg)		127.21±19.77	124.29±19.44	126.17±16.95	128.67±20.67	130.35±19.25	<0.001
DBP (mmHg)		74.57±11.53	72.35±11.36	75.47±10.23	74.33±11.08	77.65±11.41	<0.001
Hypertension stage							<0.001
	Normal	3,496 (38.8)	1,853 (46.0)	73 (35.1)	674 (37.6)	896 (30.0)	
	Prehypertension	3,360 (37.3)	1,398 (34.7)	99 (47.6)	644 (35.9)	1,219 (40.8)	
	Stage 1 hypertension	1,483 (16.5)	542 (13.5)	28 (13.5)	312 (17.4)	601 (20.1)	
	Stage 2 hypertension	676 (7.5)	236 (5.9)	8 (3.9)	163 (9.1)	269 (9.0)	
Hypertension phenotype							<0.001
	Normal	3,496 (38.8)	1,853 (46.0)	73 (35.1)	674 (37.6)	896 (30.0)	
	ISH	1,301 (14.4)	509 (12.6)	20 (9.6)	324 (18.1)	448 (15.0)	
	IDH	143 (1.6)	40 (1.0)	1 (0.5)	21 (1.2)	81 (2.7)	
	SDH	715 (7.9)	229 (5.7)	15 (7.2)	130 (7.3)	341 (11.4)	
WC (cm)		84.12±9.53	76.08±5.14	80.01±4.46	87.04±4.29	93.49±6.70	<0.001
BMI (kg/m^2^)		23.00±3.37	20.44±1.85	25.09±1.17	22.30±1.42	26.73±2.24	<0.001

Values are presented as mean±standard deviation or number (%). NWNCO, normal weight non-central obesity; NWCO, normal weight central obesity; AWNCO, abnormal weight non-central obesity; AWCO, abnormal weight central obesity; SBP, systolic blood pressure; DBP, diastolic blood pressure; ISH, isolated systolic hypertension; IDH, isolated diastolic hypertension; SDH, systolic diastolic hypertension; WC, waist circumstance; BMI, body mass index.

1 Economic status was evaluated by the natural logarithm of per capita expenditures and was divided into 3 groups based on tertiles.

**Table 2.** Obesity phenotype with hypertension stages and phenotypes in 2011^1^

Variables		Stage			Phenotype		
		Prehypertension	Stage 1 hypertension	Stage 2 hypertension	ISH	IDH	SDH
Overall (n=9,015)							
	NWNCO	1.00 (reference)	1.00 (reference)	1.00 (reference)	1.00 (reference)	1.00 (reference)	1.00 (reference)
	AWNCO	2.05 (1.50, 2.81)	1.69 (1.06, 2.69)	1.32 (0.61, 2.86)	1.49 (0.86, 2.57)	0.58 (0.08, 4.28)	2.10 (1.17, 3.78)
	NWCO	1.37 (1.20, 1.56)	1.61 (1.35, 1.92)	2.01 (1.58, 2.55)	1.63 (1.35, 1.97)	1.61 (0.93, 2.77)	1.87 (1.47, 2.39)
	AWCO	2.13 (1.89, 2.40)	3.06 (2.62, 3.57)	3.53 (2.84, 4.39)	2.58 (2.17, 3.07)	4.08 (2.72, 6.12)	4.19 (3.42, 5.13)
Male (n=4,457)							
	NWNCO	1.00 (reference)	1.00 (reference)	1.00 (reference)	1.00 (reference)	1.00 (reference)	1.00 (reference)
	AWNCO	2.54 (1.62, 3.97)	2.24 (1.23, 4.09)	1.92 (0.81, 4.55)	2.00 (1.00, 3.98)	-^2^	2.92 (1.45, 5.88)
	NWCO	1.46 (1.20, 1.79)	1.78 (1.37, 2.31)	1.95 (1.37, 2.77)	1.64 (1.23, 2.19)	2.67 (1.30, 5.48)	2.01 (1.45, 2.79)
	AWCO	2.43 (2.04, 2.91)	3.56 (2.85, 4.46)	3.89 (2.86, 5.27)	2.91 (2.25, 3.76)	5.64 (3.23, 9.85)	4.30 (3.28, 5.63)
Female (n=4,558)							
	NWNCO	1.00 (reference)	1.00 (reference)	1.00 (reference)	1.00 (reference)	1.00 (reference)	1.00 (reference)
	AWNCO	1.63 (1.03, 2.58)	1.14 (0.53, 2.45)	0.44 (0.06, 3.33)	0.95 (0.37, 2.44)	0.77 (0.10, 6.01)	1.03 (0.31, 3.45)
	NWCO	1.19 (0.99, 1.42)	1.31 (1.03, 1.68)	1.79 (1.28, 2.50)	1.48 (1.15, 1.91)	0.73 (0.31, 1.70)	1.54 (1.07, 2.23)
	AWCO	1.85 (1.57, 2.17)	2.53 (2.04, 3.16)	3.04 (2.23, 4.16)	2.29 (1.80, 2.91)	2.51 (1.42, 4.44)	3.70 (2.71, 5.05)

Values are presented as odds ratio (95% confidence interval). ISH, isolated systolic hypertension; IDH, isolated diastolic hypertension; SDH, systolic diastolic hypertension; NWNCO, normal weight non-central obesity; AWNCO, abnormal weight non-central obesity; NWCO, normal weight central obesity; AWCO, abnormal weight central obesity.

1 Odds ratios were calculated using a general logistic model, adjusting for age, sex, residence, educational level, economic status, smoking history, and alcohol consumption.

2 ‘-’ represents an insufficient sample size.

**Table 3.** Baseline characteristics of participants from 2011 to 2015 according to obesity phenotype

Characteristics		Overall (n=4,961)	NWNCO (n=2,378)	AWNCO (n=122)	NWCO (n=968)	AWCO (n=1,493)	p-value
Age (yr)		61.57±8.65	62.32±8.71	59.07±7.99	62.97±9.19	59.68±7.87	<0.001
Sex							<0.001
	Male	2,423 (48.8)	1,472 (61.9)	66 (54.1)	332 (34.3)	553 (37.0)	
	Female	2538 (51.2)	906 (38.1)	56 (45.9)	636 (65.7)	940 (63.0)	
Educational level							<0.001
	Never received any formal education	2506 (50.5)	1231 (51.8)	54 (44.3)	540 (55.8)	681 (45.6)	
	Primary school	1064 (21.5)	533 (22.4)	27 (22.1)	175 (18.1)	329 (22.0)	
	Middle school	951 (19.2)	426 (17.9)	28 (23.0)	178 (18.4)	319 (21.4)	
	High school and above	439 (8.9)	187 (7.9)	13 (10.7)	75 (7.8)	164 (11.0)	
Economic status^1^							<0.001
	Low	1497 (30.2)	768 (32.3)	34 (27.9)	316 (32.6)	379 (25.4)	
	Middle	1590 (32.1)	778 (32.7)	42 (34.4)	286 (29.6)	484 (32.4)	
	High	1874 (37.8)	832 (35.0)	46 (37.7)	366 (37.8)	630 (42.2)	
Residence							<0.001
	Rural	3411 (68.8)	1791 (75.3)	75 (61.5)	646 (66.7)	899 (60.2)	
	Urban	1550 (31.2)	587 (24.7)	47 (38.5)	322 (33.3)	594 (39.8)	
Smoking history		2112 (42.6)	1268 (53.3)	57 (46.7)	313 (32.3)	474 (31.8)	<0.001
Drinking history		1781 (35.9)	986 (41.5)	46 (37.7)	302 (31.2)	447 (29.9)	<0.001
SBP (mmHg)		124.68±18.70	122.09±18.45	126.74±17.71	126.55±19.34	127.41±18.21	<0.001
DBP (mmHg)		73.38±10.85	71.67±10.59	75.57±9.79	73.27±10.69	76.00±10.92	<0.001
WC (cm)		83.32±9.20	76.03±5.10	79.78±4.70	86.83±4.16	92.94±6.37	<0.001
BMI (kg/m^2^)		22.81±3.25	20.48±1.81	25.00±1.18	22.37±1.34	26.63±2.19	<0.001

Values are presented as mean±standard deviation or number (%). NWNCO, normal weight non-central obesity; NWCO, normal weight central obesity; AWNCO, abnormal weight non-central obesity; AWCO, abnormal weight central obesity; SBP, systolic blood pressure; DBP, diastolic blood pressure; WC, waist circumstance; BMI, body mass index.

1 Economic status was evaluated by the natural logarithm of per capita expenditures and was divided into 3 groups based on tertiles.

**Table 4.** Obesity phenotypes with hypertension stage and phenotype transitions from 2011 to 2015^1^

Variables		n	NWNCO	AWNCO	NWCO	AWCO
Stage transition^2^						
	Normal to stage 1 hypertension	516	1.00 (reference)	1.53 (0.86, 2.70)	1.21 (0.93, 1.57)	1.75 (1.40, 2.19)
	Normal to stage 2 hypertension	84	1.00 (reference)	2.29 (0.68, 7.69)	1.95 (1.11, 3.42)	1.73 (0.99, 3.01)
	Maintained stage 1 hypertension	285	1.00 (reference)	1.04 (0.37, 2.90)	1.62 (1.14, 2.29)	2.77 (2.06, 3.72)
	Maintained stage 2 hypertension	64	1.00 (reference)	1.33 (0.17, 10.21)	1.70 (0.84, 3.41)	2.80 (1.50, 5.25)
	Stage 1 hypertension to stage 2 hypertension	89	1.00 (reference)	1.78 (0.28, 11.27)	1.50 (0.76, 2.95)	0.78 (0.40, 1.50)
Phenotype transition^3^						
	Normal to ISH	409	1.00 (reference)	1.99 (1.09, 3.62)	1.39 (1.05, 1.85)	1.56 (1.20, 2.02)
	Normal to IDH	48	1.00 (reference)	-^4^	0.57 (0.21, 1.53)	1.33 (0.71, 2.51)
	Normal to SDH	143	1.00 (reference)	1.53 (0.54, 4.35)	1.27 (0.75, 2.14)	2.54 (1.72, 3.75)
	ISH to SDH	67	1.00 (reference)	-^4^	1.62 (0.75, 3.51)	0.92 (0.44, 1.93)
	IDH to SDH	13	1.00 (reference)	-^4^	-^4^	-^4^

Values are presented as odds ratio (95% confidence interval). NWNCO, normal weight non-central obesity; AWNCO, abnormal weight non-central obesity; NWCO, normal weight central obesity; AWCO, abnormal weight central obesity; ISH, isolated systolic hypertension; IDH, isolated diastolic hypertension; SDH, systolic diastolic hypertension.

1 Odds ratios were calculated using a general logistic model, adjusting for age, sex, residence, educational level, economic status, smoking history, and alcohol consumption.

2 The “stage 1 hypertension to stage 2 hypertension” group used the “maintained stage 1 hypertension” group as the reference, while the other groups of changes in hypertension stages used the “maintained normal” group as the reference.

3 The “ISH to SDH” group used the “maintain ISH” group as the reference, the “IDH to SDH” group used the “maintained IDH” group as the reference, and the other groups used the “maintained normal” group as the reference.

4 ‘-’ represents an insufficient sample size.

## SUPPLEMENTARY MATERIALS

Supplementary Material 1. OR (95% CI) of greater BMI and excessive WC with stages and phenotypes of hypertension in 2011 stratified by sex

Supplementary Material 2 OR (95% CI) of WC with transitions of stages and phenotypes of hypertension stratified by body type and of BMI with transitions of stages and phenotypes of hypertension stratified by WC from 2011 to 2015

Supplementary Material 3 Forest plot of the associations between obesity phenotypes and hypertension stages in longitudinal analysis. AWNCO, abnormal weight non-central obesity; NWCO, normal weight central obesity; AWCO, abnor mal weight central obesity; OR, odds ratio; CI, confidence interval.

Supplementary Material 4 Forest plot of the associations between obesity phenotypes and hypertension phenotypes in the logitudinal analysis. AWNCO, ab- normal weight non-central obesity; NWCO, normal weight central obesity; AWCO, abnormal weight central obesity; SDH, systolic diastolic hypertension; IDH, isolated diastolic hypertension; ISH, isolated systolic hypertension; OR, odds ratio; CI, confidence interval.

Supplementary Material 5 OR (95% CI) of obesity phenotypes with hypertension stage and phenotype transitions from 2013 to 2015
