# Supplementary material for: The associations of obesity phenotypes with the risk of hypertension and its transitions among middle-aged and older Chinese adults
Source: Epidemiol Health. 2023 Apr 10;45:e2023043. doi: 10.4178/epih.e2023043 (PMC10593582; doi:10.4178/epih.e2023043)
Supplement: Supplementary Material 1. — OR (95% CI) of greater BMI and excessive WC with stages and phenotypes of hypertension in 2011 stratified by sex [file epih-45-e2023043-Supplementary-1.docx]

**Supplement**

Greater BMI and excessive WC were associated with all hypertension stages and phenotypes (Supplementary Material 1). Furthermore, larger WC and BMI at baseline was found to be associated with transitions of hypertension stage and phenotype (Supplementary Material 2). Furthermore, larger WC was associated with hypertension stage transitions in people with abnormal weight than in those with normal weight, while the larger BMI was associated with hypertension stage transitions and phenotype transitions in normal WC than excessive WC.

Supplementary Materials 3 and 4 visualized the Associations of obesity phenotypes with the transitions of hypertension stages and phenotypes from 2011 to 2015.

The association of greater WC with hypertension was found to be more significant among those with greater BMI, whereas the association of greater BMI with hypertension was found to be more significant among those with normal WC.

Supplementary Material 1. OR (95% CI) of greater BMI and excessive WC with stages and phenotypes of hypertension in 2011 stratified by sex

|  | **Stage transition** | | |  | **Phenotype transition** | | |
| --- | --- | --- | --- | --- | --- | --- | --- |
|  | **Prehypertension** | **Stage 1 hypertension** | **Stage 2 hypertension** |  | **ISH** | **IDH** | **SDH** |
| ***Overall(N=9015)*** |  |  |  |  |  |  |  |
| Overweight | **1.78 (1.59-1.99)** | **2.19 (1.88-2.54)** | **2.14 (1.74-2.63)** |  | **1.87 (1.58-2.21)** | **3.24 (2.23-4.72)** | **2.59 (2.13-3.14)** |
| Obesity | **2.72 (2.23-3.32)** | **4.18 (3.28-5.32)** | **4.69 (3.43-6.41)** |  | **3.20 (2.42-4.24)** | **4.36 (2.49-7.65)** | **6.41 (4.88-8.44)** |
| Excessive WC | **1.83 (1.64-2.03)** | **2.53 (2.20-2.90)** | **2.94 (2.44-3.56)** |  | **2.18 (1.87-2.54)** | **3.77 (2.64-5.37)** | **3.33 (2.79-3.98)** |
| ***Male(N=4457)*** |  |  |  |  |  |  |  |
| Overweight | **2.10 (1.76-2.50)** | **2.68 (2.15-3.34)** | **2.55 (1.88-3.46)** |  | **2.23 (1.73-2.89)** | **4.01 (2.40-6.69)** | **2.90 (2.22-3.79)** |
| Obesity | **3.31 (2.31-4.73)** | **5.17 (3.40-7.85)** | **6.69 (4.01-11.16)** |  | **4.38 (2.70-7.12)** | **3.97 (1.62-9.70)** | **7.08 (4.53-11.07)** |
| Excessive WC | **2.02 (1.69-2.40)** | **2.93 (2.37-3.64)** | **3.31 (2.49-4.40)** |  | **2.48 (1.94-3.18)** | **5.02 (3.07-8.20)** | **3.46 (2.69-4.46)** |
| ***Female(N=4558)*** |  |  |  |  |  |  |  |
| Overweight | **1.56 (1.34-1.81)** | **1.86 (1.52-2.28)** | **1.85 (1.40-2.45)** |  | **1.66 (1.33-2.07)** | **2.51 (1.46-4.31)** | **2.24 (1.70-2.96)** |
| Obesity | **2.46 (1.92-3.14)** | **3.66 (2.70-4.96)** | **3.77 (2.51-5.66)** |  | **2.73 (1.92-3.88)** | **4.15 (2.00-8.59)** | **5.79 (4.05-8.27)** |
| Excessive WC | **1.67 (1.45-1.92)** | **2.19 (1.83-2.63)** | **2.56 (1.99-3.29)** |  | **1.97 (1.62-2.40)** | **2.64 (1.60-4.35)** | **3.05 (2.37-3.93)** |

Note: WC, Waist circumstance. ISH, Isolated systolic hypertension, IDH, Isolated diastolic hypertension, SDH, Systolic diastolic hypertension. OR, odds ratio. CI, confidence interval. OR values were conducted by general logistic model, adjusting for age, gender, residence, educational level, economic status, smoking history, alcohol.
