# Supplementary material for: The associations of obesity phenotypes with the risk of hypertension and its transitions among middle-aged and older Chinese adults
Source: Epidemiol Health. 2023 Apr 10;45:e2023043. doi: 10.4178/epih.e2023043 (PMC10593582; doi:10.4178/epih.e2023043)
Supplement: Supplementary Material 2 — OR (95% CI) of WC with transitions of stages and phenotypes of hypertension stratified by body type and of BMI with transitions of stages and phenotypes of hypertension stratified by WC from 2011 to 2015 [file epih-45-e2023043-Supplementary-2.docx]

**Supplement**

Greater BMI and excessive WC were associated with all hypertension stages and phenotypes (Supplementary Material 1). Furthermore, larger WC and BMI at baseline was found to be associated with transitions of hypertension stage and phenotype (Supplementary Material 2). Furthermore, larger WC was associated with hypertension stage transitions in people with abnormal weight than in those with normal weight, while the larger BMI was associated with hypertension stage transitions and phenotype transitions in normal WC than excessive WC.

Supplementary Materials 3 and 4 visualized the Associations of obesity phenotypes with the transitions of hypertension stages and phenotypes from 2011 to 2015.

The association of greater WC with hypertension was found to be more significant among those with greater BMI, whereas the association of greater BMI with hypertension was found to be more significant among those with normal WC.

Supplementary Material 2. OR (95% CI) of WC with transitions of stages and phenotypes of hypertension stratified by body type and of BMI with transitions of stages and phenotypes of hypertension stratified by WC from 2011 to 2015

|  |  | **Stage** | | | | |  |  | **Phenotype** | | | | |
| --- | --- | --- | --- | --- | --- | --- | --- | --- | --- | --- | --- | --- | --- |
|  | N | Normal to Stage 1 hypertension | Normal to Stage 2 hypertension | Maintained Stage 1 hypertension | Maintained Stage 2 hypertension | Stage 1 hypertension to Stage 2 hypertension |  | N | Normal to ISH | Normal to IDH | Normal to SDH | ISH to SDH | IDH to SDH |
| **WC** |  |  |  |  |  |  |  |  |  |  |  |  |  |
| Overall | 4872 | **1.42 (1.21-1.67)** | 1.27 (0.87-1.87) | **2.07 (1.67-2.55)** | **2.39 (1.55-3.69)** | 1.13 (0.70-1.82) |  | 4784 | **1.28 (1.07-1.54)** | 1.44 (0.87-2.38) | **1.76 (1.32-2.35)** | 0.66 (0.39-1.12) | - |
| Normal weight | 3290 | 1.16 (0.88-1.53) | 1.12 (0.60-2.08) | **1.82 (1.25-2.65)** | 1.84 (0.88-3.87) | 1.91 (0.85-4.30) |  | 3253 | 1.17 (0.87-1.57) | 1.16 (0.47-2.86) | 1.04 (0.59-1.83) | 1.28 (0.55-2.99) | - |
| Abnormal weight | 1582 | 1.22 (0.86-1.73) | 1.26 (0.53-3.03) | **1.68 (1.10-2.55)** | **2.85 (1.13-7.16)** | 1.01 (0.34-3.00) |  | 1531 | 1.08 (0.72-1.64) | 1.80 (0.66-4.93) | 1.40 (0.80-2.43) | **0.08 (0.02-0.37)** | - |
| **BMI** |  |  |  |  |  |  |  |  |  |  |  |  |  |
| Overall | 4872 | **1.30 (1.18-1.44)** | 1.10 (0.86-1.41) | **1.56 (1.37-1.77)** | **1.74 (1.34-2.26)** | 1.02 (0.78-1.32) |  | 4784 | **1.21 (1.07-1.35)** | 1.15 (0.84-1.56) | **1.53 (1.28-1.82)** | 0.84 (0.61-1.16) | - |
| Normal WC | 3314 | **1.27 (1.06-1.52)** | 1.16 (0.77-1.74) | **1.59 (1.22-2.05)** | 1.25 (0.70-2.23) | 1.21 (0.66-2.22) |  | 3274 | **1.25 (1.03-1.53)** | 0.77 (0.43-1.38) | **1.45 (1.03-2.04)** | 1.07 (0.56-2.05) | - |
| Excessive WC | 1558 | 1.22 (1.00-1.50) | 1.04 (0.61-1.77) | **1.25 (1.01-1.54)** | 1.52 (0.98-2.37) | 0.80 (0.52-1.22) |  | 1510 | 1.13 (0.89-1.44) | 1.29 (0.72-2.32) | 1.40 (1.00-1.96) | 0.64 (0.37-1.12) | - |

Note: WC, Waist circumstance. BMI, Body mass index, ISH, Isolated systolic hypertension, IDH, Isolated diastolic hypertension, SDH, Systolic diastolic hypertension. OR, odds ratio. CI, confidence interval. The unit of BMI was standard deviation. OR values were conducted by a total general logistic model, adjusting for age, gender, residence, educational level, economic status, smoking history, alcohol consumption. ‘ - ’ represents insufficient sample size. Group ‘Stage 1 hypertension to Stage 2 hypertension’ take group ‘Maintain Stage 1 hypertension’ as reference, other groups of changed hypertension stages take group ‘Maintained Normal’ as reference. Group ‘ISH to SDH’ take group ‘Maintain ISH’ as reference; group ‘IDH to SDH’ take group ‘Maintain IDH’ as reference, other groups take group ‘Maintain Normal’ as reference.
