# Supplementary material for: The associations of obesity phenotypes with the risk of hypertension and its transitions among middle-aged and older Chinese adults
Source: Epidemiol Health. 2023 Apr 10;45:e2023043. doi: 10.4178/epih.e2023043 (PMC10593582; doi:10.4178/epih.e2023043)
Supplement: Supplementary Material 3 — Forest plot of the associations between obesity phenotypes and hypertension stages in longitudinal analysis. AWNCO, abnormal weight non-central obesity; NWCO, normal weight central obesity; AWCO, abnor mal weight central obesity; OR, odds ratio; CI, confidence interval. [file epih-45-e2023043-Supplementary-3.docx]

**Supplement**

Greater BMI and excessive WC were associated with all hypertension stages and phenotypes (Supplementary Material 1). Furthermore, larger WC and BMI at baseline was found to be associated with transitions of hypertension stage and phenotype (Supplementary Material 2). Furthermore, larger WC was associated with hypertension stage transitions in people with abnormal weight than in those with normal weight, while the larger BMI was associated with hypertension stage transitions and phenotype transitions in normal WC than excessive WC.

Supplementary Materials 3 and 4 visualized the Associations of obesity phenotypes with the transitions of hypertension stages and phenotypes from 2011 to 2015.

The association of greater WC with hypertension was found to be more significant among those with greater BMI, whereas the association of greater BMI with hypertension was found to be more significant among those with normal WC.


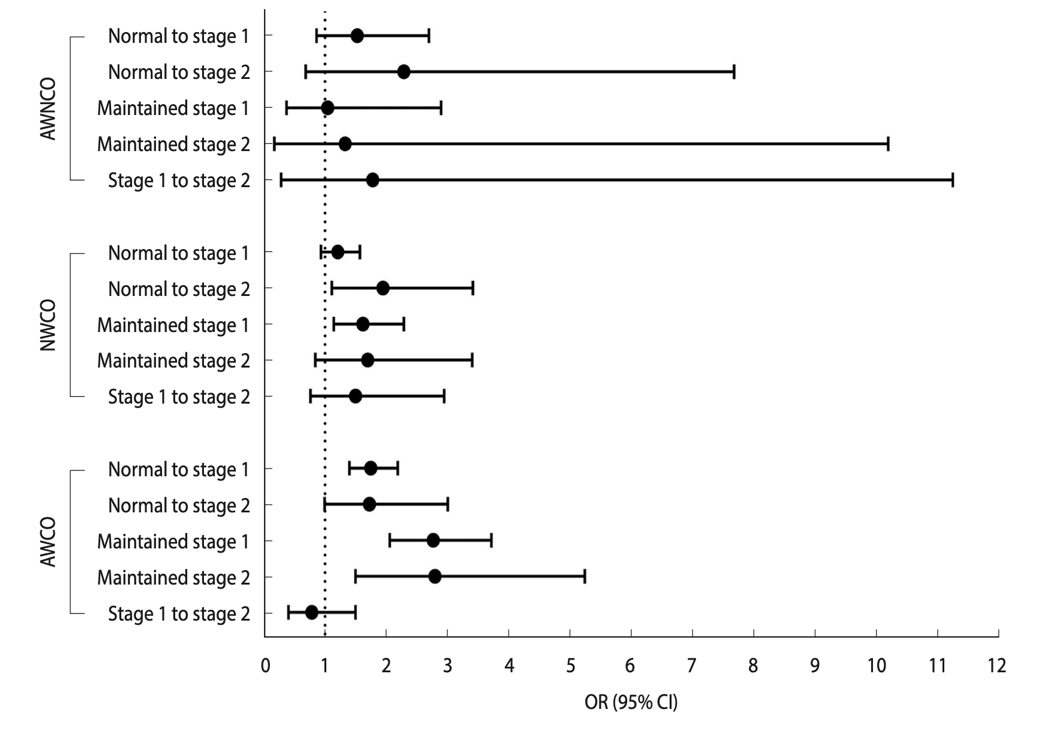
**Supplementary Material 3.** Forest plot of the associations between obesity phenotypes and hypertension stages in longitudinal analysis. AWNCO, abnormal weight non-central obesity;

NWCO, normal weight central obesity; AWCO, abnor mal weight central obesity; OR, odds ratio; CI, confidence interval.
